# Hypertriglyceridaemia and the risk of pancreatitis six months post lopinavir/ritonavir initiation

**DOI:** 10.4102/sajhivmed.v19i1.766

**Published:** 2018-06-26

**Authors:** Wilhelm P. Greffrath, Jesslee M. du Plessis, Michelle Viljoen, Marike Cockeran

**Affiliations:** 1 KwaZulu-Natal Department of Health, South Africa; 2 Medicine Usage in South Africa (MUSA), North-West University, South Africa; 3 Centre of Excellence for Pharmaceutical Sciences, Division of Pharmacology, Faculty of Health Sciences, North-West University, South Africa; 4 Pharmacology and Clinical Pharmacy, School of Pharmacy, Faculty of Natural Sciences, University of the Western Cape, South Africa

## Abstract

**Background:**

Hypertriglyceridaemia (HTG) is an important risk factor for pancreatitis and cardiovascular disease (CVD), depending on severity. Hypertriglyceridaemia is common in human immunodeficiency virus (HIV) infection and is also a common complication of lopinavir/ritonavir (LPV/r).

**Objectives:**

To evaluate the risk of pancreatitis associated with HTG in patients six months post initiation of LPV/r-based therapy in a regional public hospital.

**Methods:**

Triglyceride (TG), serum amylase (s-amylase) and CD4+ count values were retrospectively investigated six months post LPV/r-based initiation. Age, gender, previous antiretroviral regimen and period since HIV diagnosis were also recorded.

**Results:**

The final sample consisted of 194 patients, 50 males and 144 females; mean (± standard deviation [s.d.]) age was 39.52 (± 9.98) years, and the mean (± s.d.) period since HIV diagnosis was 91.32 (± 25.18) months. Normal TG levels (< 1.70 mmol/L) were detected in only 55% of patients and the rest presented with some degree of HTG. The mean (± s.d.) TG for the entire sample was elevated at 1.94 (± 1.30) mmol/L with the mean (± s.d.) of the males at 2.36 (± 1.74) – statistically higher compared to the females at 1.79 (± 1.08) mmol/L (*p* = 0.034). No cases of pancreatitis were recorded and the time since HIV diagnosis did not indicate any statistically significant differences in the means of the TG, serum amylase or CD4 count values.

**Conclusion:**

Triglyceride levels were not substantially elevated to induce pancreatitis at six months post initiation of LPV/r, but were elevated above the accepted upper normal limit of 1.70 mmol/L which may have implications for cardiovascular risk.

## Introduction

Significant advancements in the understanding and treatment of the human immunodeficiency virus (HIV) have changed the prognosis of HIV infection from a fatal disease to a chronic illness.^1^ The prolonged life expectancy of the HIV-infected population elucidated not only the long-term pathophysiologic effects of the disease itself but also the long-term side effects of antiretroviral therapy (ART).^2^ Infection with HIV itself causes comorbidities unrelated to acquired immunodeficiency syndrome (AIDS) such as cardiovascular, renal and bone disease, as well as diabetes mellitus and pancreatitis. The long-term side effects of ART include loss of renal function, loss of bone density, insulin resistance and dyslipidaemia with saquinavir, indinavir, nelfinavir and lopinavir/ritonavir (LPV/r) that are well known for increasing triglycerides (TG).^2,3^ Pancreatitis and atherosclerosis are both associated with hypertryglyceridaemia (HTG) – HTG is a proven cause of pancreatitis, but thus far only linked to atherosclerosis.^4,5,6^ Because HIV infection has no cure, and consequently its therapy is lifelong, the effects of each on, *inter alia*, the pancreas and cardiovascular system become important in order to correctly manage and treat the related risks that may arise in the long term.^2,7^ Management of HTG-associated risk factors and comorbidities should include not only the reactive treatment but also the aggressive prevention thereof.^2^

The LPV/r combination is useful in decreasing the probability of the development of viral resistance.^8,9,10,11,12^ A well-documented metabolic side effect of LPV/r is increased TG.^3,13^ Related metabolic side effects of LPV/r include hypercholesterolaemia, hyperglycaemia and insulin resistance.^3^ Hypertriglyceridaemia is a known cause of pancreatitis.^14,15^ Although TG-induced pancreatitis is not the main aetiology of pancreatitis, it is not uncommon,^16^ with as many as 10% of cases of acute pancreatitis because of HTG.^17^

Triglyceride plasma concentrations above 1.7 mmol/L, 2.3 mmol/L and 5.6 mmol/L are classified as mild, moderate and severe, respectively,^5,18^ with very severe HTG above 11.3 mmol/L.^4^ Triglyceride values of > 20 mmol/L are usually because of a combination of primary and secondary factors.^19^ The main consequences of HTG are increased risk of pancreatitis and atherosclerosis.^4,6^ The risk for pancreatitis seems to increase with an increase in TG,^15^ but only becomes a significant concern at values > 11.3 mmol/L.^4,5,20^

In acute pancreatitis, serum amylase and serum lipase (s-amylase and s-lipase, respectively) are elevated three times higher than the upper normal limit.^21,22^ The upper normal limit for this study was 2.08 µkat/L (104 U/L as defined by National Health Laboratory Service [NHLS]).

At mild to moderate HTG, the risk for cardiovascular disease (CVD) becomes more prevalent.^18^ Hypertriglyceridaemia is a characteristic sign of atherogenic dyslipidaemia,^23^ but the exact manner of contribution of TG to cardiovascular risk is still unclear.^5,6^ It is not clear whether there is a causal relationship between HTG and atherosclerosis,^15^ but it is now accepted that TG is at least indirectly associated with cardiovascular risk.^6^

The focus of this study was on the effects of the combination of LPV/r on TG levels and to assess the risk of TG-induced pancreatitis six months post initiation.

## Methods

This was a cross-sectional, retrospective, observational study and was conducted at a public sector hospital of approximately 500 beds. The following data were collected from patients’ medical history: age, gender, s-amylase, TG, CD4+ count, period since date of HIV diagnosis to present and prior antiretroviral regimens. Data used from the pharmacy dispensing statistics indicated that as of August 2015, 807 adult patients received LPV/r-based treatment from the hospital. A power analysis based on chi-square analysis of association indicated that a minimum sample size of 197 was required for an effect size of at least 0.2. The TIER.Net system (previously known as the HIV electronic register or eRegister) is a database of all patients receiving ART countrywide and is maintained by the National Department of Health (NDoH). Simple random sampling was used to select 200 patients from the TIER.Net database. The database numbered each of the patients on the list that represented the study population. The head of department at the Centre for Disease Control (CDC) then generated random numbers that fell in the numerical range of the list. For each number that was generated, a patient was selected and the process repeated until the required sample size was achieved. The data were retrospectively collected during April 2016 by two medical officers permanently appointed at the CDC of the hospital.

The CDC, a department within this hospital, is dedicated to the management and treatment of patients suffering from HIV and/or acquired immune deficiency syndrome (AIDS) on an outpatient basis. All adult Black patients older than 18 years who were on LPV/r-based treatment that accessed the services of the CDC at any time in the past with all of TG, s-amylase and CD4+ results six months post LPV/r initiation were eligible to be included. If this information was not available, it was regarded as incomplete. The selection process therefore had to be repeated every time a patient with incomplete data was selected.

This study population was kept of homogenous Black race by design in order to rule out genetic confounders as both Raza and coworkers^24^ and Riedel and coworkers^25^ indicated that pancreatitis was highest among HIV-infected African-Americans. Most factors or pre-existing diseases that may independently increase TG (familial HTG, acute hepatitis, nephrotic syndrome)^18^ or precipitate pancreatitis (azathioprine, pentamidine, stavudine, valproic acid, tetracycline, metronidazole, isoniazid, dapsone, tamoxifen, oestrogen, alcohol consumption)^22^ were excluded where possible. Hypothyroidism and diabetes mellitus were not excluded. Medication that may induce or inhibit the metabolism of LPV/r (such as ketoconazole and antiepileptic agents), fibrates or other cholesterol-lowering agents were excluded. The use of alcohol could not be reliably excluded unless recent consumption was recorded in the patients’ medical histories.

Non-fasting TG values had to be used because fasting values were not available. Perceived disadvantages of non-fasting lipid assays are that the values are less standard and less accurate and that it is unclear at which level intervention is warranted.^26^ However, because of the meals and snacks normally consumed during the day, lipid values are at a constant state of non-fasting. Non-fasting lipid values therefore offer a better measurement of the risk of CVD.^26^ The median non-fasting TG values are usually slightly more elevated than fasting TG values. However, fasting TG values are only indicated when non-fasting values are above 5 mmol/L.^26^ Fasting TG levels should therefore be taken with the clinical context in mind.

Descriptive statistics mean (standard deviation [± s.d.]) and median (interquartile range [IQR]) were calculated for all the variables for the entire sample and separately for males and females. An independent *t*-test was performed to investigate a possible significant difference in TG, s-amylase and CD4 counts between males and females. In addition, the duration since HIV diagnosis was categorised to investigate any differences in the means of the TG, s-amylase and CD4 counts. The three categories chosen (0–79 months, 80–104 months and > 104 months) were to ensure the same number of participants in each group (*n* = 65, 65 and 64). Statistical significance was set at *p* < 0.05, and practical significance was tested with Cohen’s *d*-value (0.2 = small effect, 0.5 = medium effect and 0.8 = large effect).

## Ethical consideration

This study was approved by the HREC-NWU on 14 March 2016 (NWU-00356-15-A1) and also by the KwaZulu-Natal Department of Health’s Directorate of Health Research and Knowledge Management (KZ_2015RP22_110), and by the Medical Manager at the facility where the study was conducted.

## Results

The TG, s-amylase and CD4 counts for 194 out of the sample of 200 patients were statistically analysed, as six patients had to be removed from the original sample because they were already on LPV/r-based regimens for more than six months.

Table 1 reflects the demographic details of 144 (74%) female and 50 (26%) male patients. The mean (± s.d.) age for the entire sample was 39.52 (± 9.98) years. The mean (± s.d.) period for all the patients since first diagnosis of HIV infection until the time of data collection was 91.32 (± 25.18) months. All the patients were on antiretroviral treatment before the rollout of the fixed drug combination of tenofovir/emtricitabine/efavirenz and were therefore still taking individually formulated drugs. The patients were on a previous regimen consisting of stavudine (d4T) or tenofovir (TDF) or zidovudine (AZT) plus lamivudine (3TC) plus efavirenz (EFV) or nevirapine (NVP). The majority of patients were on a stavudine-based combination (*n* = 157, 81.93%), and since 2010, stavudine was phased out and replaced with tenofovir (*n* = 36, 18.56%).

**TABLE 1:** Demographics and previous regimens.

Variable	Female	Male	Total
*n*	144	50	194
Mean age (± s.d.)	38.87 (± 9.23)	41.40 (± 11.77)	39.52 (± 9.98)
Median age (IQR)	38.00 (33.00–42.00)	41.50 (37.50–46.50)	38.00 (34.00–44.25)
Mean months (± s.d.) since HIV diagnosis	90.08 (± 25.45)	94.92 (± 24.29)	91.32 (± 25.18)
**Previous regimen frequency**			
d4T/3TC/EFV	59	32	91
d4T/3TC/NVP	61	5	66
TDF/3TC/EFV	7	13	20
TDF/3TC/NVP	16	0	16
AZT/3TC/NVP	1	0	1
**Total**	**144**	**50**	**194**

s.d., standard deviation; IQR, interquartile range; 3TC, lamivudine; AZT, zidovudine; d4T, stavudine; EFV, efavirenz; NVP, nevirapine, TDF, tenofovir.

Table 2 summarises the mean (± s.d.) values of TG, s-amylase and CD4+ count of the female, male and total group and also indicates the statistical and practical significance between the two genders. The mean (± s.d.) TG was 1.94 (± 1.30) mmol/L, and the median (IQR) for the whole population was 1.48 (1.35) mmol/L. The mean (± s.d.) TG values for females and males were 1.79 (± 1.08) mmol/L and 2.36 (± 1.74) mmol/L, respectively, which was statistically significant (*p* = 0.034).

**TABLE 2:** Results of plasma variables at six months post lopinavir/ritonavir initiation.

Variable	Female (*n* = 144)				Male (*n* = 50)				*t*-test		Total (*n* = 194)			

	Mean	± s.d.	Median	IQR	Mean	± s.d.	Median	IQR	p*	Cohen’s *d*†	Mean	± s.d.	Median	IQR
TG mmol/L	1.79	± 1.08	1.44	1.10–2.14	2.36	± 1.74	1.89	0.93–3.72	0.034*	0.330	1.94	± 1.30	1.48	1.05–2.40
S-amylase µkat/L	2.19	± 0.90	1.99	1.58–2.54	2.30	± 1.01	2.08	1.58–2.77	0.463	0.11	2.22	± 0.93	2.04	1.58–2.63
CD4 × 10^3^/L	392	± 222	361	237–517	287	± 226	232	125–389	0.004*	0.47	365	± 227	328	200–483

s.d., standard deviation; IQR, interquartile range; TG, triglyceride.

* , *p* < 0.05 statistically significant.

† , Cohen’s *d* (practical significance) 0.2 = small effect; 0.5 = medium effect; 0.8 = large effect.

**TABLE 3:** Difference between mean and mean-ranked values per time period since human immunodeficiency virus diagnosis.

Mean	Variable	0–79 months (*n* = 65)		80–104 months (*n* = 65)		> 104 months (*n* = 64)		*p*

		Mean	± s.d.	Mean	± s.d.	Mean	± s.d.	
Values per time period (± s.d.)	**One-way ANOVA:**							
	TG mmol/L	1.83	± 1.14	1.92	± 1.25	2.07	± 1.50	0.591
	S-amylase µkat/L	2.11	± 0.82	2.26	± 1.07	2.29	± 0.88	0.495
	CD4 × 10^3^/L	349.15	± 236.07	381.29	± 243.74	363.82	± 200.37	0.723
Rank values per period	**Kruskal–Wallis:**							
	TG mmol/L	95.18	-	95.41	-	101.98	-	0.738
	S-amylase µkat/L	92.07	-	95.77	-	104.77	-	0.418
	CD4 × 10^3^/L	91.71	-	100.43	-	100.41	-	0.594

ANOVA, analysis of variance; s.d., standard deviation; TG, triglyceride.

Only 1% of the patients in this study had severe HTG values of above 5.6 mmol/L. A further 26% had moderate HTG with values between 2.3 mmol/L and 5.6 mmol/L. Mild HTG with values between 1.7 mmol/L and 2.3 mmol/L occurred in 19% of the patients. The remaining 55% were all below 1.7 mmol/L. Although the TG levels were taken at random without fasting and may therefore render slightly higher TG values, a significant total of 45% of the patients had some degree of HTG with 42% of females and 54% of the male population presenting with HTG.

The criterion for pancreatitis for this study was an elevation of s-amylase three times above the upper normal limit of 2.08 µkat/L. No cases of pancreatitis were recorded after six months of LPV/r treatment; the mean (± s.d.) s-amylase value for the entire sample was 2.22 (± 0.93) µkat/L and the median (IQR) value was 2.04 (± 1.05) µkat/L. There was a small and insignificant difference (*p* = 0.463) in the mean (± s.d.) s-amylase values between female patients (2.19 [± 0.90] µkat/L) and male patients (2.31 [± 1.01] µkat/L).

The mean (± s.d.) CD4 count for the entire sample after the first six months of LPV/r-based therapy was 365 (± 227) cells/mm^3^. There was a statistical (*p* = 0.004) as well as a practical significant difference (0.47) in the mean (± s.d.) CD4 counts between the female and male patients [392 (± 222) cells/mm^3^ and 287 (± 226) cells/mm^3^, respectively]. The elapsed time since the date of diagnosis of the HIV infection until the date of data collection was divided into three categories (0–79 months, 80–104 months and > 104 months). Variation between the mean values and mean rank values for TG, s-amylase and CD4 counts for each time period was investigated. Even though there was an increase in mean and mean rank values of TG and s-amylase as a function of time, there was no statistically significant difference between any of the values from the corresponding time periods.

## Discussion

The results from this study suggest that the risk for TG-induced pancreatitis after the first six months of LPV/r is improbable because no case of acute pancreatitis was recorded. None of the TG values recorded were > 11.3 mmol/L in order to possibly induce pancreatitis. In fact, the mean TG value was only 1.94 mmol/L. For the purposes of assessing the risk of HTG-induced pancreatitis in this population, the difference between a fasting and a non-fasting TG value is not relevant because the mean TG value was only 1.94 mmol/L – well below 11.33 mmol/L. However, outliers above 5 mmol/L would warrant a fasting value to serve as a baseline to monitor those patients with a genetic predisposition for HTG.^26^ Even though no cases of pancreatitis were identified according to the criteria of this study, the s-amylase values showed a tendency to be increased above the upper normal limit at the six-month mark with some individual values close to reaching the value requisite for pancreatitis for this study. A total of 3.1% (*n* = 6) of the patients had s-amylase values above double the upper normal value. However, according to Oliveira and coworkers^7^ and Manfredi and coworkers,^27^ post-mortem evidence of pancreatitis is very common in the HIV-infected population. Many cases of pancreatitis may therefore be subclinical and may go unnoticed.^27^ Given the low mean TG value of this study sample, if there were cases of undetected subclinical pancreatitis, they were probably not because of HTG. It is estimated that pancreatitis pathology is as much as 800 times more common in the HIV-infected population than otherwise,^20^ which this study could not corroborate. Asymptomatic elevations in s-amylase and s-lipase are quite common, but cases of acute pancreatitis are not frequently reported among HIV-infected patients.^28^ Oliveira and coworkers^7^ confirmed that drug-induced pancreatitis may therefore be frequently overlooked when mild but clinically meaningful increases in s-amylase and s-lipase values are present. Considering that several other factors may precipitate pancreatitis in an HIV-infected population, physicians should therefore be suspicious of mildly elevated s-amylase values, as was suggested by Oliveira and coworkers,^7^ especially in an uncontrolled environment where any combination of risk factors may be present.

Factors such as age, gender and ethnicity affect TG values.^4^ Triglyceride values are normally higher in males than in females,^4^ and there was a statistically significant difference between the means of males and females. The mean TG value for males was a notable 32% higher than that of the females. This raises questions regarding the susceptibility of males compared to females to the HTG-inducing effects of LPV/r.

The difference in mean s-amylase levels between males and females was small and insignificant, suggesting that neither gender was more susceptible to pancreatitis than the other in this study. However, Oliveira and coworkers^7^ and Riedel and coworkers^25^ both found female gender to be a risk factor for pancreatitis among HIV-infected patients, especially those of lower body weight. In both these studies, they cited several studies with very large sample sizes of several thousand participants, and the study durations were also longer than six months. A possible shortcoming of this study was the relatively short period of observation (six months) and population size. A much larger population may therefore be required to better determine the risk of pancreatitis in a given population.

Where CD4 counts are concerned, it has been well established that lower CD4 counts correlate with higher risk of pancreatitis.^7,25^ This study found the males to have significantly lower mean CD4 counts compared to females, which puts them at a higher risk of pancreatitis. This is contrary to the findings of both Oliveira and coworkers^7^ and Riedel and coworkers^25^ regarding the risk contributed by gender. However, there is evidence that males access medical care later^29^ and could thus have lower CD4+ counts because of commencing ART later compared to females.

Furthermore, in the HIV clinical cohort study conducted by Riedel and coworkers^25^ to investigate the risk factors for pancreatitis of the cases of acute pancreatitis recorded during the period of their study, ethnicity was found to be a risk factor in a bivariate analysis with an odds ratio of 1.99 (*p* = 0.058). This would suggest that ethnicity could be an influencing factor. The median population age in the study by Riedel and coworkers^25^ was 36 years, which was similar to the median age of 38 years for this study. This suggests that the population investigated for this study may also carry more risk of acute pancreatitis than not only the general population but perhaps also other HIV-positive populations of different ethnicity.

In this study, a significant number of 45% of the patients had some degree of HTG, which may largely be attributed to LPV/r therapy, because factors that may independently increase TG values were excluded from the sample as far as possible. This was slightly lower than the results of Calza,^3^ who found HTG in 60% – 90% of patients on protease inhibitors. Baseline TG values will therefore be important in order to quantify the exact impact of LPV/r therapy on TG values, which were not available for this study.

Other risk factors include the total period infected with HIV (mean of 91.3 months in this study population) and a CD4 count of less than 200 cells/mm^3^.^7^ In this study, the comparative mean values of TG, s-amylase and CD4 did not significantly vary over the three consecutive categorical periods of HIV infection duration. However, even though statistically insignificant, the mean values of TG and s-amylase did increase over the three categorical periods, which may suggest that proactive monitoring of these parameters would be wise and may warrant further investigation.

### Study limitations

The lack of baseline values for TG, s-amylase and CD4 counts meant that the effect of LPV/r on the change of these variables could not be quantified. In addition, the TG values were taken randomly and were not after fasting. The mild HTG may therefore be overestimated. Body mass index values and dietary habits (including alcohol consumption) were also not consistently and reliably available, which could influence TG values. The more specific s-lipase instead of s-amylase would have enabled a more accurate diagnostic criterion for cases of pancreatitis. Patients with hypothyroidism and diabetes mellitus were not excluded from the sample. The observation period of six months was too short to predict the long-term effects of LPV/r-induced HTG and possibly pancreatitis; therefore, future studies should be expanded to include, at minimum, 12 months duration on LPV/r-based regimens.

## Conclusion and recommendations

Almost half (45%) of the patients in this study had mild elevated TG values after the first six months of LPV/r treatment. Triglyceride values were not clinically elevated to cause a realistic risk of pancreatitis. However, comorbidities and drugs that increase TG in uncontrolled circumstances should not be disregarded. Severe HTG was rare, but mild to moderate HTG was more common. Mild to moderate HTG is linked to cardiovascular risk (but no causal effect yet established), starting at values lower than required to cause pancreatitis. A considerable part of the study population may therefore be at risk of developing CVD. The current guidelines from the NDoH^30^ for adults also recommend a fasting TG and cholesterol assay to be done at baseline when LPV/r therapy is indicated, then only repeated again after three months of LPV/r therapy. This approach will identify the patients most at risk during the formative stages of LPV/r-based therapy. No baseline TG values were available on the TIER.Net database. The magnitude and rate with which TG values increased could therefore not be established. However, as Calza and co-workers demonstrated,^3^ the HTG-inducing effect of LPV/r is time-dependent and TG values can more than double from three months post initiation of LPV/r therapy to 12 months post initiation. Further investigation is therefore warranted to better predict the risks of HTG beyond the first three months of LPV/r-based therapy. Because many cases of pancreatitis are subclinical and may therefore be unnoticed,^27^ it may be of value to test s-amylase and s-lipase 12 monthly in larger longitudinal studies.
